# Hyperoxia resensitizes chemoresistant human glioblastoma cells to temozolomide

**DOI:** 10.1007/s11060-012-0923-3

**Published:** 2012-07-05

**Authors:** Stella Sun, Derek Lee, Nikki P. Lee, Jenny K. S. Pu, Stanley T. S. Wong, W. M. Lui, C. F. Fung, Gilberto K. K. Leung

**Affiliations:** Division of Neurosurgery, Department of Surgery, Li Ka Shing Faculty of Medicine, University of Hong Kong, Queen Mary Hospital, 102 Pokfulam Road, Hong Kong, Hong Kong

**Keywords:** Glioma, Temozolomide, Chemoresistance, Hypoxia, Apoptosis, MAPK

## Abstract

Temozolomide (TMZ) is standard chemotherapy for glioblastoma multiforme (GBM). Intratumoral hypoxia is common in GBM and may be associated with the development of TMZ resistance. Oxygen therapy has previously been reported to potentiate the effect of chemotherapy in cancer. In this study, we investigated whether hyperoxia can enhance the TMZ-induced cytotoxicity of human GBM cells, and whether and how it would resensitize TMZ-resistant GBM cells to TMZ. TMZ-sensitive human GBM cells (D54-S and U87-S) were treated with TMZ to develop isogenic subclones of TMZ-resistant cells (D54-R and U87-R). All cell lines were then exposed to different oxygen levels (1, 21, 40, or 80 %), with or without concomitant TMZ treatment, before assessment of cell cytotoxicity and morphology. Cell death and survival pathways elicited by TMZ and/or hyperoxia were elucidated by western blotting. Our results showed that TMZ sensitivity of both chemo-sensitive and resistant cells was enhanced significantly under hyperoxia. At the cell line-specific optimum oxygen concentration (D54-R, 80 %; U87-R, 40 %), resistant cells had the same response to TMZ as the parent chemosensitive cells under normoxia via the caspase-dependent pathway. Both TMZ and hyperoxia were associated with increased phosphorylation of ERK p44/42 MAPK (Erk1/2), but to a lesser extent in D54-R cells, suggesting that Erk1/2 activity may be involved in regulation of hyperoxia and TMZ-mediated cell death. Overall, hyperoxia enhanced TMZ toxicity in GBM cells by induction of apoptosis, possibly via MAPK-related pathways. Induced hyperoxia is a potentially promising approach for treatment of TMZ-resistant GBM.

## Introduction

Glioblastoma multiforme (GBM) is a highly malignant primary brain tumor characterized by rapid growth, invasiveness, early recurrence, and resistance to conventional therapy. Overall prognosis is poor; median survival is approximately 12 months [1]. The current regimen for treatment of GBM after maximum surgical resection includes concomitant chemo-radiation using temozolomide (TMZ), followed by six cycles of adjuvant TMZ [2]. Although TMZ can significantly prolong survival, many patients continue to suffer from recurrent disease, because of de-novo or acquired drug resistance [3, 4]. The mechanism underlying TMZ resistance is incompletely understood [5], and, because TMZ is now standard therapy and forms the control arm in clinical trials of other novel agents, understanding and overcoming TMZ resistance is urgent, and is an active area of investigation [6].

The biological effects of hyperoxia have been widely studied for a variety of neurological conditions, for example carbon monoxide poisoning, traumatic brain injury, and ischemic stroke [7–9]. The potential beneficial effects of hyperoxia have been attributed to increased oxygenation within the plasma and brain tissues which can potentially stabilize intracranial pressure, prevent blood–brain barrier disruption, suppress neutrophil–endothelial adhesion, and reduce edema [8, 10]. On the other hand, hyperoxia may activate apoptosis by induction of reactive oxygen species (ROS). Hyperoxia may also interfere with expression of cytokines, growth factors, and transcription factors whereby survival or cell-death pathways are affected to different degrees [11]. Its therapeutic role, therefore, remains controversial.

Intra-tumoral hypoxia is common in GBM, and hypoxic cancer cells are known to be more resistant to radiation or cytotoxic drugs [12, 13]. Conversely, hyperoxia has been shown to potentiate the effect of these treatments by enhancing chemocytotoxicity in vitro [14] and neovascularization in vivo [15]. Although hyperoxia has previously been described for control of tumor growth and progression in glioma, its potential application as an adjunct to chemotherapy has not been investigated [16, 17]. In this study we investigated whether and how different concentrations of environmental oxygen would affect TMZ toxicity in chemosensitive and chemoresistant GBM cells. The hypothesis was that hyperoxia would enhance the effect of TMZ especially in TMZ-resistant GBM cells.

## Materials and methods

### TMZ-resistant cells and treatment

We have previously described the development of isogenic subclones of TMZ-resistant GBM cells by means of chronic TMZ exposure [18]. Briefly, human GBM cell lines, D54-MG (Duke University Medical Center, USA) and U87-MG (American Type Culture Collection, Manassas, VA, USA), were cultured in Dulbecco’s modified Eagle’s medium (DMEM)/F12 (1:1) and minimum essential medium (MEM)-α, respectively. These were supplemented with 10 % heat inactivated fetal bovine serum (Gibco; Invitrogen, Grand Island, NY, USA). Parent TMZ-sensitive cells (designated D54-S and U87-S) were initially exposed to 100 μM TMZ (Temodal; Schering–Plough, Whitehouse Station, NJ, USA) for two weeks and then continuously to the IC_50_ of TMZ for 12 months. The TMZ-resistant subclones (D54-R and U87-R) so produced were isolated and maintained in low dose (100 μM) TMZ.

### Oxygen treatment

TMZ-sensitive (D54-S and U87-S) and resistant (D54-R and U87-R) cells were exposed to different degrees of oxygen saturation under normobaric conditions (hypoxia, 1 %; normoxia, 21 %; hyperoxia 40 or 80 %), with or without concomitant TMZ for 72 h. Cells were then examined for in-vitro chemosensitivity, apoptosis-related and survival-related protein changes.

### Clonogenic assay

Cells were seeded at a density of 500 cells/well on six-well plates. After incubation for 24 h they were treated with TMZ (250, 500, 1,000, or 2,000 μM) for 48 h. Cells without TMZ challenge were used as control cells. Cells were then rinsed with fresh medium and left to form colonies after incubation for 14 days. Colonies were then stained with crystal violet (5 g/L; Sigma–Aldrich, Saint Louis, MO, USA). Percentage inhibition was calculated as the number of colonies formed under the treatment conditions relative to the control. Error bars represented standard deviations from three independent experiments conducted in triplicate. **P < 0.01 and *P < 0.05, versus control.

### Cytotoxicity assay

Cytotoxicity was measured by sulforhodamine B (SRB) assay (Sigma–Aldrich). Cells were seeded in 96-well plates (5,000 cells/well) and cultured in the absence or presence of TMZ (250, 500, 1,000, 2,000, and 3,000 μM) for 96 h. Determination of cell density was based on cellular protein content as measured by absorbance (OD) at 490 nm. The percentages of viable cells relative to the controls (cells without previous TMZ treatment) were calculated and plotted. The IC_50_ values were calculated by derivation of the best-fit line, by use of three independent experiments performed in triplicate.

### Western blot

Total protein lysates (30 μg) were separated by 12 % SDS-PAGE using a Mini-Protean electrophoresis cell (Bio-Rad Laboratories, Hercules, CA, USA), and then transferred to a PVDF membrane (0.22 µm; Millipore, Bedford, MA, USA) by use of a Mini Trans-Blot cell (Bio-Rad Laboratories). After blocking, the membrane was probed with one of the following primary antibodies at 1:1,000 dilution for 1 h: rabbit monoclonal antibodies against caspase 3, bax, bcl-2, total p44/42 MAP Kinase (Erk 1/2), and phosphor-p44/42 MAP kinase (Erk1/2) (Thr202/Tyr204) (all from Cell Signaling Technology, Danvers, MA, USA). HRP-conjugated secondary antibodies at 1:10,000 were then used for incubation for 1 h. Immunoreactivity signals were amplified by use of the ECL Plus western blotting detection system (GE Biosciences, Buckinghamshire, UK).

## Results

### TMZ-resistant GBM cells

**Fig. 1:**
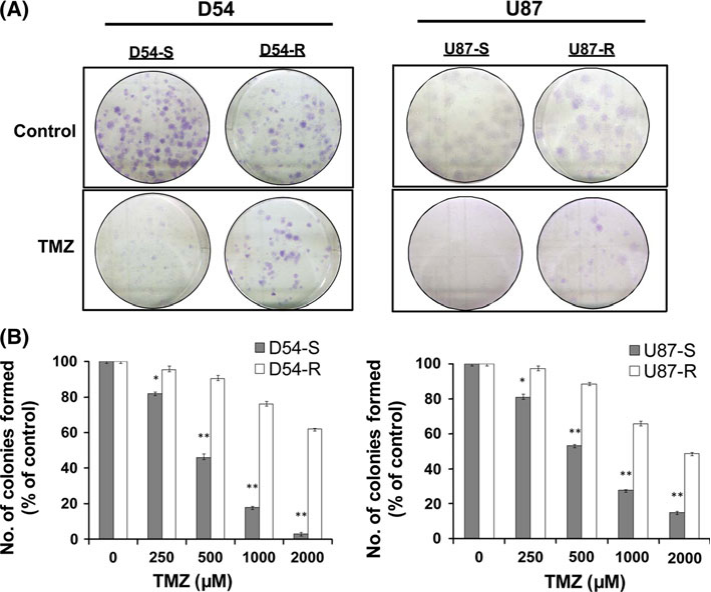
TMZ-resistant D54-R and U87-R cells. (**a**) Representative dishes from clonogenic assay of the four cells 14 days after TMZ treatment. (**b**) Clonogenic percentage survival of TMZ-sensitive (D54-S and U87-S) and TMZ-resistant (D54-R and U87-R) cells after rechallenge with TMZ. Each experiment was repeated three times and performed in triplicate. *Columns*, mean results from triplicate assay; *bars*, SD; *P < 0.05 and **P < 0.01

After exposing the parent D54-S and U87-S cells to TMZ for 10 months, isogenic TMZ-resistant subclones (D54-R and U87-R) were developed. Clonogenic formation assays were performed after TMZ re-challenge. Compared with the resistant cell lines (D54-R and U87-R), reduced clonogenic formation was observed for D54-S and U87-S 14 days after TMZ treatment (Fig. 1a). Colony formation was significantly inhibited (*P* < 0.05) in the TMZ-sensitive cell lines upon further challenge with different concentrations of TMZ (250 500, 1,000, and 2,000 μM) (Fig. 1b). On the basis of the percentages of colonies formed relative to the control, IC_50_ for D54-R and U87-R cells was increased sixfold (IC_50_ = 2,861.6 μM; *P* < 0.05) and threefold (IC_50_ = 1,731.74 μM; *P* < 0.05) compared with the parent control cells (D54-S, IC_50_ = 480.8 μM and U87-S, IC_50_ = 590.1 μM). The results indicated that we had successfully generated two isogenic TMZ-resistant GBM subclones.

### Hyperoxia induced cell death synergistically with TMZ

**Fig. 2:**
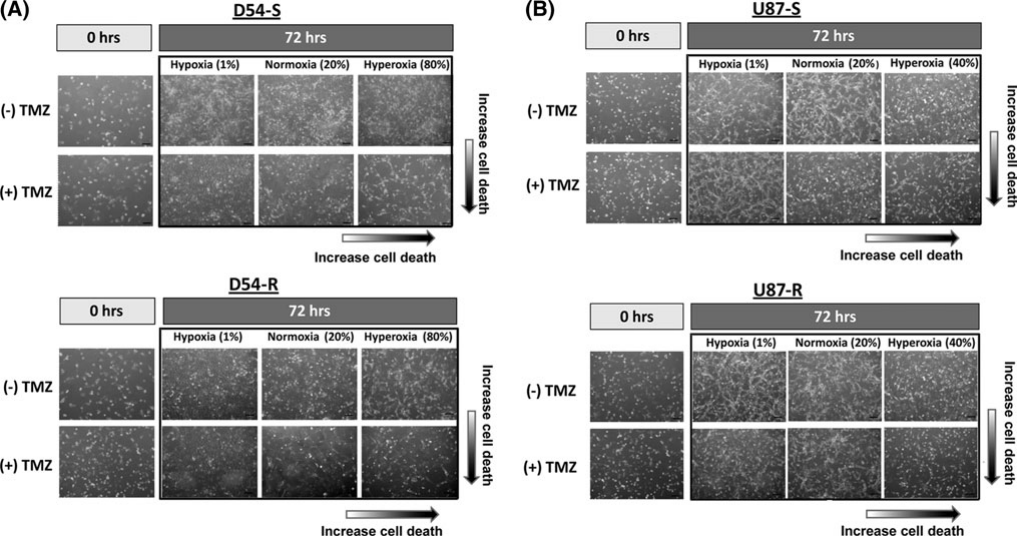
Morphology changed after 72 h treatment at different oxygen saturation with or without concomitant TMZ. **a** D54, **b** U87 cells

Seeded cells were incubated for 72 h at three oxygen levels (hypoxia, 1 %; normoxia, 21 %; and hyperoxia, 40 or 80 %) with or without concomitant TMZ. In the absence of TMZ, survival of both chemosensitive (D54-S and U87-S) and chemoresistant (D54-R and U87-R) cells increased under hypoxia (1 %), and a mild decrease was observed under cell type-dependent optimum hyperoxic conditions (D54-80 % and U87-40 %) (Fig. 2). With concomitant TMZ given at the IC_50_ for the parent cells, hyperoxia caused a marked decrease in cell survival, especially for the TMZ-resistant cells. These findings were suggestive of a synergistic effect between hyperoxia and TMZ in inducing cell death. Furthermore, under light microscopy, cells cultured under hyperoxia had altered morphology with increased pseudopod formation (Fig. 2).

### Hyperoxia enhances chemosensitivity of TMZ-resistant GBM cells

**Fig. 3:**
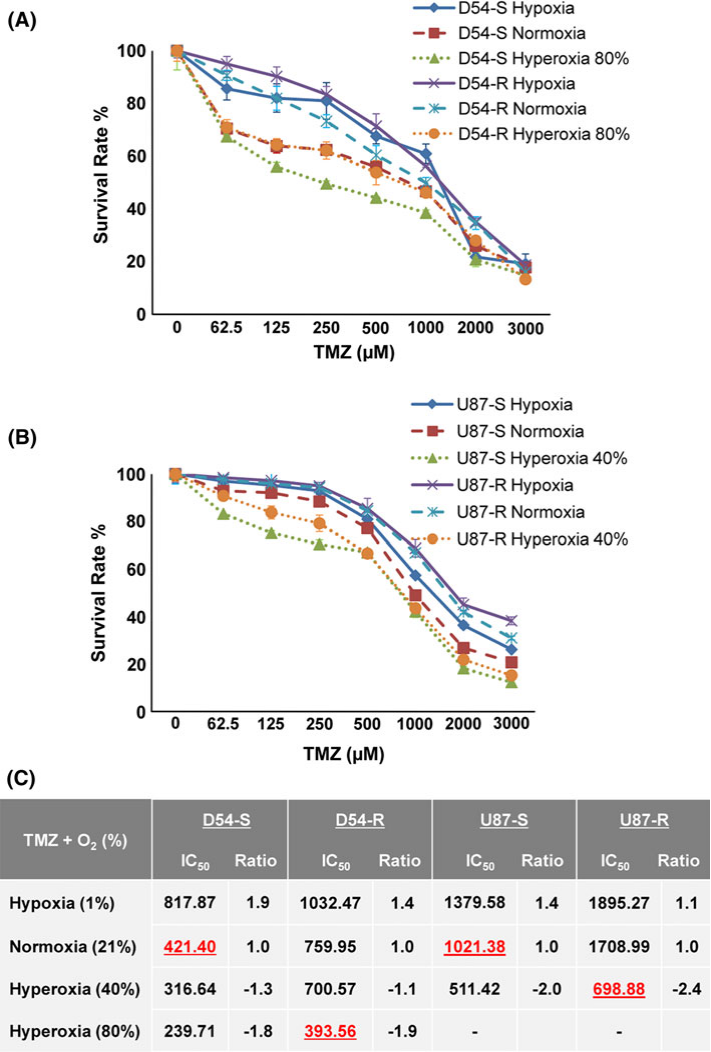
Survival curves for **a** D54 and **b** U87 cells under different oxygen saturation conditions. **c** Alteration of TMZ IC_50_ values and their respective ratio to normoxia under different oxygen saturation conditions for TMZ-sensitive (D54-S and U87-S) and TMZ-resistant (D54-R and U87-R) cells

In-vitro chemosensitivity assay showed that cell survival was affected by the ambient oxygen concentration. Figure 3a, b shows that hypoxia had a survival benefit for both sensitive (D54-S and U87-S) and resistant (D54-R and U87-R) cells against TMZ, whereas hyperoxia led to growth inhibition. Figure 3c shows the corresponding IC_50_ values and their respective ratios compared with the IC_50_ values under normoxia. Overall, IC_50_ were highest for both TMZ-sensitive and TMZ-resistant cell lines under hypoxia. Compared with cells under normoxia, the IC_50_ for D54-S and D54-R cells increased 1.9-fold and 1.4-fold, respectively, under hypoxia. Similarly, the IC_50_ for U87-S and U87-R cells increased 1.4-fold and 1.1-fold, respectively. Hyperoxia had the opposite effect, resulting in a rapid reduction in cell growth. At 40 % hyperoxia, 1.3 and 2-fold decreases in IC_50_ were observed for D54-S and U87-S cells, respectively. Similar inhibitory effects were also observed for D54-R and U87-R cells, for which 1.1 and 2.4-fold decreases, respectively, were observed.

This phenomenon of hyperoxia-induced growth inhibition was cell line-dependent (Fig. 3c). D54 cells were less sensitive to 40 % oxygen than U87 cells, as was apparent from the lower ratio compared with normoxia for both sensitive and resistant cells. At 80 % hyperoxia, 1.8 and 1.9-fold decreases in IC_50_ were observed for D54-S and D54-R cells, respectively, whereas most of the U87 cells died (results not shown). Moreover, hyperoxia was found to ameliorate TMZ resistance in both D54-R and U87-R cells, with a decreases in IC_50_ from 759.95 μM (D54-R) and 1708.99 μM (U87-R) under 21 % O_2_, to 393.56 μM (D54-R under 80 % O_2_) and 698.88 μM (U87-R under 40 % O_2_), respectively. These were lower than that for the parent cell lines under normoxic conditions. Our findings indicate that the response of GBM to TMZ was significantly affected by environmental oxygen saturation. Hyperoxia may potentiate the anti-tumor effect of TMZ, whereas hypoxia may reduce TMZ sensitivity.

### Hyperoxia-induced cell death through caspase-dependent pathways

**Fig. 4:**
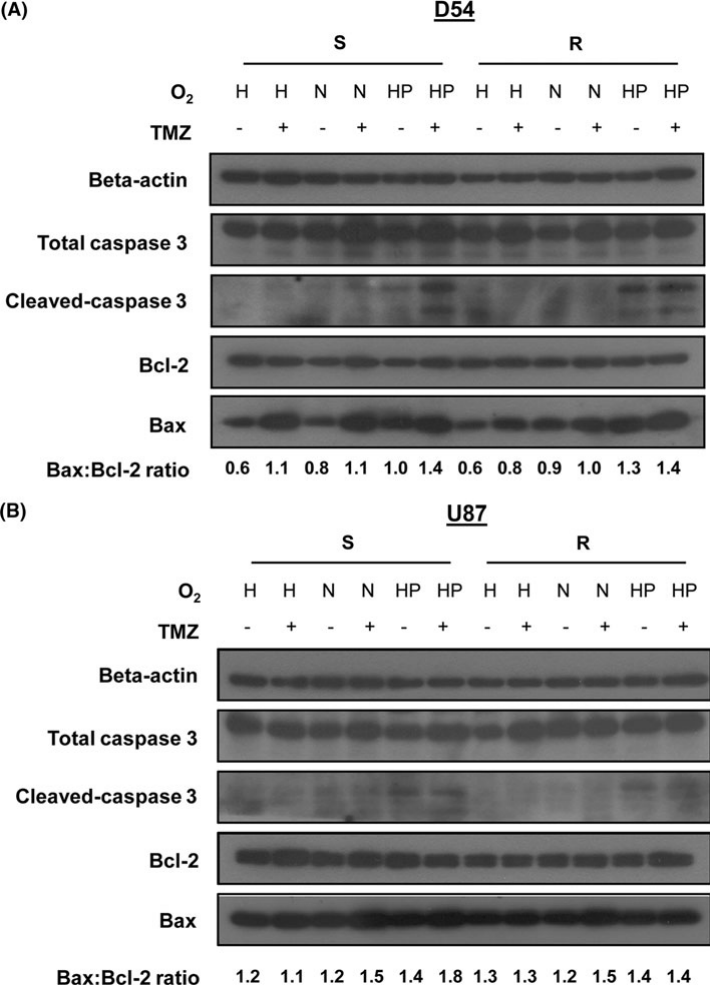
Western blotting shows upregulation of apoptotic mediators. Activation of caspase 3 and increased Bax:Bcl-2 ratio are positively associated with increased oxygen saturation and concomitant TMZ. **a** D54 and **b** U87. *HP*, hyperoxia; *N*, normoxia; *H*, hypoxia; *S*, TMZ-sensitive GBM cells; *R*, TMZ-resistant GBM cells

We then examined if hyperoxia-induced cell death was dependent on pro-apoptotic pathways. Mediators of apoptosis were examined for their altered protein expression under different treatment conditions. Treatment with TMZ or exposure to hyperoxia alone was associated with upregulation of the pro-apoptotic protein Bax, and almost unaltered, if not reduced, expression of the anti-apoptotic protein Bcl-2 (Fig. 4). These resulted in an increased Bax:Bcl-2 ratio, hence cell death. The most significant response was observed after combining TMZ with hyperoxia, which resulted in an increase in caspase 3 activity, known to be the “executioner” of apoptosis. Our findings suggested that the synergistic effects of TMZ and hyperoxia were at least partially mediated via caspase-dependent apoptosis. Interestingly, for D54-R and U87-R cells activation of apoptosis was of similar extent after exposure to hyperoxia with or without concomitant TMZ. The result suggested that hyperoxia alone may be potent enough for attenuation of TMZ resistance.

### Hyperoxia activated the ERK p44/42 MAPK (Erk1/2) signal-transduction pathway

**Fig. 5:**
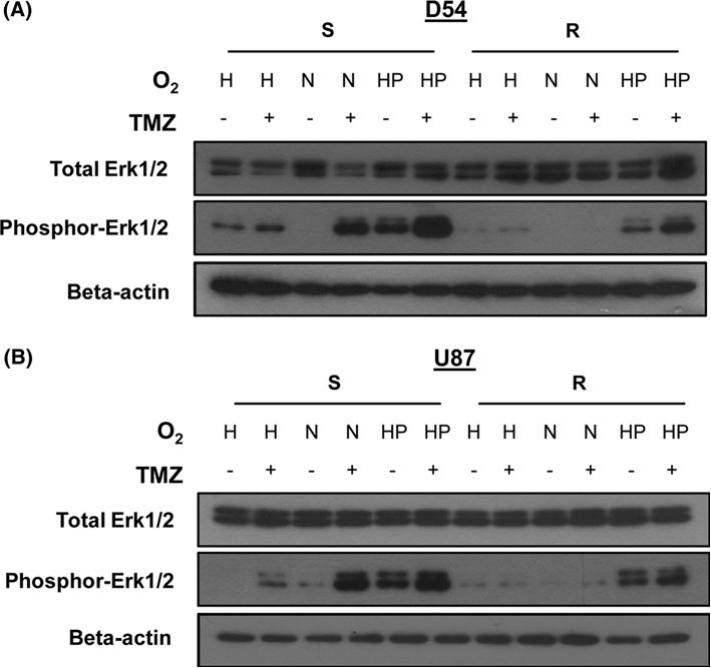
Activation of the Erk1/2 MAPK signal transduction pathway after exposure to hyperoxia and TMZ treatment. Increased phosphorylation of Erk1/2 proteins under hyperoxia and concomitant TMZ conditions as demonstrated by western blotting. **a** D54 and **b** U87

Cellular stress, for example DNA damage, and oxidative stress are known to activate cascades of protein kinases that participate in signal transduction. The MAPK pathway is one of these signaling pathways. We therefore investigated whether activation of ERK1/2 was involved in GBM’s responses to hyperoxia and TMZ. We immunoblotted the total and phosphorylated forms of Erk1/2 proteins under different treatment conditions (Fig. 5). We found upregulation of phosphor-Erk1/2 protein in both the TMZ-sensitive (D54-S and U87-S) and TMZ-resistant (D54-R and U87-R) cells after hyperoxia or TMZ treatment, indicating that both stressful stimuli could activate the Erk1/2 signal transduction pathway. Moreover, Erk1/2 activity was higher in D54-S and U87-S cells than in D54-R and U87-R cells after 72 h, suggesting that TMZ-resistant GBM cells may be less responsive to oxidative and DNA damage stressors. We then investigated the effect of combined hyperoxia–TMZ treatment. For both D54-S and U87-S cells, phosphor-Erk1/2 expression increased in an oxygen-dependent manner when coupled with TMZ treatment (Fig. 5). The findings suggested the MAPK pathway may be important in the hyperoxia-induced and TMZ-induced signal transduction which mediates other death signaling pathways.

## Discussion

TMZ, an imidazotetrazine derivative of the alkylating agent dacarbazine, is currently standard therapy for GBM. TMZ is well-tolerated and is therapeutically beneficial because of its ability to methylate DNA at the N-7 or O-6 positions of guanine residues. Despite the positive effect of TMZ on survival, treatment failure because of drug resistance is a significant clinical issue. Several mechanisms underlying TMZ resistance in GBM have been described, including the expression of the repair enzyme *O*-6-methylguanine-DNA-methyltransferase (MGMT) [19, 20], loss of p53 function [21], selection of pre-existing TMZ-resistant cells [3], mitochondrial adaptive response [22], and dysregulation of glucose transporters and drug metabolism [23]. At present, effective strategies for treatment of TMZ-resistant GBM are lacking. Use of *O*-6-benzylguanine (O6-BG), an inhibitor of MGMT, has been tested in a phase II trial, with mixed outcomes [24].

GBM is characterized by poorly organized vasculature, hypoxic areas, and necrosis. Hypoxia in tumors is generally associated with increased aggressiveness and resistance to chemotherapy and radiation [12]. In GBM, hypoxia is associated with increased expression of stem-cell markers, and formation of neurospheres and other drug-resistant phenotypes [25]. Pastolatto et al. reported high expression of MGMT (a DNA repair enzyme in GBM stem cells) that was positively correlated with intra-tumoral hypoxia and TMZ resistance [26]. In an attempt to overcome TMZ resistance, inhibition of hypoxia-inducible factors (HIFs), a key regulator of cellular responses to hypoxia, has been conceived as a therapeutic approach for enhancement of chemosensitivity [27, 28]. Inhibitors of HIFs have been studied as chemotherapy adjuncts with therapeutic agents, for example angiogenesis inhibitors and cytotoxic drugs [29, 30]. Shen et al. reported the robust anti-tumor effects of combining HIF-1α inhibition with TMZ in D54 GBM cells [31].

We have adopted a different approach to modulation of hypoxia-mediated chemoresistance—use of induced hyperoxia. Hyperoxia has previously been shown to inhibit cell proliferation [32] and potentiate the effect of chemotherapy by enhancement of cytotoxicity or neovascularization in cancers [33]. Its use as a chemotherapy adjunct has been described for a variety of conditions, for example ovarian [15], breast [34], bone [35], prostate [36], and lung cancers [37]. For brain tumors, hyperoxia in the form of hyperbaric oxygen therapy (HBO) has been shown to increase oxygen level both within and around glioma tissues in patients [38]. Hyperoxia may also promote a reversion from anaerobic metabolism to non-tumorigenic oxidative metabolism [39]. Reported studies on HBO for brain tumors focused mainly on its role as a radiation sensitizer [40, 41] and in the prevention of radiation-induced brain injury [42]. The potential use of hyerpoxia as a chemotherapy adjunct remains unexplored. Stuhr et al. reported reduced tumor growth, reduced vascular density, and changes in gene expression after normobaric and moderate HBO therapy but without concomitant TMZ [16].

As far as we are aware, ours is the first study using hyperoxia as an adjuvant to TMZ therapy for GBM. With an in-vitro model, we demonstrated that normobaric hyperoxia could enhance TMZ cytotoxicity whereas hypoxia had survival benefits. Furthermore, TMZ-resistant cells could regain the same chemo-sensitivity toward TMZ as their parent TMZ-sensitive cells. The potential clinical implication is that hyperoxia may be used to attenuate TMZ resistance in GBM. Regarding the underlying mechanism, our findings suggested that hyperoxia-induced apoptosis was likely to be caspase-dependent. Caspase activation is the irreversible onset of apoptosis [43], and several studies have also described caspase activation as one of the crucial steps in hyperoxia-induced cell death [44, 45]. Our study provides additional information about this phenomenon in brain tumor cells, in that caspase 3 activation was enhanced synergistically under combined hyperoxia–TMZ treatment. Similar changes in Bax:Bcl-2 ratio were also indicative of the importance of pro and anti-apoptotic proteins [46].

We also demonstrated that Erk1/2 MAPK may be critically involved in regulating hyperoxia-induced cell death [47]. The latter may be regulated by a variety of factors [43], for example activation of the protein kinases that participate in signal transduction [48]. Although it is generally believed to be a survival mediator for protecting cells against cell death, Erk1/2 MAPK has been shown to mediate hyperoxic cell death [49, 50]. In our study, the increased phosphorylation of Erk1/2 after exposure to concomitant hyperoxia–TMZ treatment was suggestive of a potentiating effect on cell death. Taken together, our findings indicate that apoptosis and MAPK pathways may be intimately involved in a signaling network under hyperoxia in GBM. The exact mechanism by which this could modulate hyerpoxia-mediated and TMZ-mediated cell death deserves further studies.

This is the first study to investigate the effect of hyperoxia as an adjunct to TMZ in GBM. Combined hyperoxia can significantly potentiate the anti-tumor effect of TMZ. Hyperoxia may attenuate TMZ resistance in GBM via MAPK signaling and apoptotic pathways. Our findings lay the foundation for further in-vivo studies, and have potentially important implications in the treatment of GBM and other cancers in which chemoresistance has a major effect on treatment outcome.

## Abbreviation

GBM: Glioblastoma multiforme
HBO: Hyperbaric oxygen
IC_50_: Half-maximum inhibitory concentration
ROS: Reactive oxygen species
SRB: Sulforhodamine B
TMZ: Temozolomide
